# What matters to people aged 80 and over regarding ambulatory care? A systematic review and meta-synthesis of qualitative studies

**DOI:** 10.1007/s10433-021-00633-7

**Published:** 2021-08-21

**Authors:** Angélique Herrler, Helena Kukla, Vera Vennedey, Stephanie Stock

**Affiliations:** 1 Faculty of Human Sciences and Faculty of Medicine, Graduate School GROW, Gerontological Research on Well-being, University of Cologne, Albertus-Magnus-Platz, 50923 Cologne, Germany; 2 Institute for Health Economics and Clinical Epidemiology, University Hospital Cologne, 50924 Cologne, Germany

**Keywords:** Aged, 80 and over, Patient-centered care, Ambulatory care, Qualitative research, Patient preferences, Systematic review

## Abstract

The growing percentage of the population aged 80 and over is challenging for healthcare systems, as frailty and other complex health issues are common in this age group. In order to provide patient-centered ambulatory healthcare, their preferences and expectations need to be explored. Therefore, the aim of this study was to systematically search for and synthesize qualitative evidence on how people aged 80 and over believe ambulatory healthcare (medical and nursing care) should be delivered to them. Medline, PsycINFO, CINAHL, Web of Science Core Collection and Google Scholar were searched for full research reports of qualitative studies focusing on the preferences, wishes, needs, expectations and experiences of people aged 80 and over regarding ambulatory medical and nursing care. The results were screened by two independent reviewers using a two-step approach. The included studies were meta-synthesized using Thomas and Harden’s ‘thematic synthesis’ approach in order to gain a new, second-order interpretation of the findings of the primary studies. In the intermediate synthesis step, 14 aspects of healthcare structures and care relationships were identified as relevant. Based on these, three underlying wishes were found: feeling safe, feeling like a meaningful human being, and maintaining control and independence. The results of this review are in line with other research, such as reviews focusing on the preferences of the younger age group (65–80). However, the importance of aspects of care relationships as an integral part of favorable ambulatory healthcare and the wish to be strengthened as a meaningful human being are emphasized more strongly.

**Supplementary Information:**

The online version contains supplementary material available at 10.1007/s10433-021-00633-7.

## Introduction

The United Nations (2019) estimate that by 2050, the number of people aged 80 and over will triple to 143 million globally. For these people, the “oldest old,” an important aspiration is ageing in place. This means to enable older people to continue living in their home and known environment with as low dependency levels as possible and therefore, avoid institutionalization and transition to a nursing home (Houben 2001). Older people show a preference for ageing in place to maintain their relationships and autonomy (Costa-Font et al. 2009; WHO 2015b; Wiles et al. 2012).

However, this is particularly challenging due to health-related impairments (Betini et al. 2017; Hajek et al. 2015). Although the effects of ageing vary between individuals, research conducted in recent years indicates a considerable deterioration in physical health status among very old people. In addition to higher multimorbidity among this age group, frailty becomes increasingly common (Collard et al. 2012; Marengoni et al. 2011; Rockwood et al. 2011; Rosero-Bixby and Dow 2009). Frailty, a state of general vulnerability, is the result of decreased capacities in different body systems interacting (Fried et al. 2004; WHO 2015b). It increases the risk of further geriatric syndromes that are highly prevalent from the age of 80, such as falls and urinary incontinence (Inouye et al. 2007; WHO 2015b). Subsequently, these older people need help with one or more aspects of daily life, and are frequent users of the healthcare system (Marengoni et al. 2011; van den Bussche et al. 2011; WHO 2015b).

Since a considerable proportion of the ageing population’s health issues are complex and chronic in nature, the purpose of healthcare services is expected to shift from acute care and curing toward (1) chronic care and (2) individualized goals and encouraging active involvement of patients. There are two major propositions for reorienting healthcare to address this shift. The first proposition is a stronger focus on ambulatory care, i.e., the provision of a broad range of healthcare services including prevention, curation and rehabilitation on an outpatient basis (Berman 2000). Ambulatory care is of particular importance for ageing in place and considered to best manage the requirements of complex and chronic care, especially regarding timely access, care coordination and cost-efficiency (WHO 2015a). Secondly, models of patient-centered care (PCC) are frequently proposed to encourage individualized care, and are now being called for by important international organizations such as the World Health Organization (2015b). In contrast to episode-based clinical care, where patients are rather passive, the core of these models is to actively involve patients and incorporate their individual values, needs and preferences (Lusk and Fater 2013; Scholl et al. 2014). As such, in order to bring healthcare systems in line with the demographic shift, older people’s subjective needs and preferences, especially regarding ambulatory care, should be explored and used as the basis for adaptations.

Previous approaches to examining older people’s healthcare needs and preferences have focused mainly on the 60 and over age group. Although people aged 80 and over were not excluded in these reviews, most participants were below 80 years of age (Gonzalez et al. 2019; Gregory et al. 2017; Holm et al. 2013; McGilton et al. 2018). Therefore, the results may not be representative for the oldest-old, especially regarding their special health issues as mentioned above. Furthermore, previous studies have focused on institutional settings such as hospitals and nursing homes (Bridges et al. 2010; Maurer et al. 2019). This issue was addressed by a recent scoping review that focused on home environments, but only included studies on home healthcare recipients (Dostálová et al. 2020). As such, the variety of healthcare needs and preferences, including those for older people who are in good enough health to manage on their own or with help of relatives in the ambulatory setting, remains unclear. To fill this research gap, a broader systematic review covering the subjective perspective of people aged 80 and over regarding ambulatory care is needed. Thus, the aim of this study was to synthesize qualitative evidence on the design of ambulatory care as desired by people aged 80 and over. The overall research question was: *What matters to people aged 80 and over regarding ambulatory care?*

## Methods

A systematic literature search and a thematic synthesis of the findings were conducted in order to provide a meta-synthesis. Qualitative meta-synthesis aims to transform the findings into integrated descriptions and explanations of the qualitative research phenomenon (Sandelowski and Barroso 2007). For this review, Thomas and Harden’s (2008) inductive “thematic synthesis” approach that focuses on the integration of individual experiences and perspectives was applied.

The review was prospectively registered at PROSPERO (record number: CRD42020158107). Reporting is based on the Preferred Reporting Items for Systematic Reviews and Meta-Analyses (PRISMA, Moher et al. 2009) and the Enhancing Transparency in Reporting the Synthesis of Qualitative Research statement (ENTREQ, Tong et al. 2012).

### Search strategy

In order to develop the search strategy, the following review question was formulated based on the overall research question: *What are the preferences, needs and expectations of the oldest-old regarding ambulatory care, from their perspective?* Since these terms are often used inconsistently or interchangeably in qualitative studies on healthcare structures, they were found to be appropriate to represent “what patients want from their healthcare” following Street et al. (2012, p. 168). Combined search terms and controlled vocabulary relating to people aged 80 and over, preferences and ambulatory medical and nursing care were used. The search was limited to qualitative studies because the area of interest was the subjective perspective of the older persons. The search strategy was piloted in PubMed, together with the inclusion criteria. The final strategy (Online Resource 1) was adapted to the other databases.

The first author searched electronic bibliographic databases related to medicine and health sciences (Medline via PubMed, PsycINFO, CINAHL, Web of Science Core Collection) for full primary research reports from inception to October 2019. A search in Google Scholar and a forward and backward citation search of included studies were also conducted. Research reported in English, German and Dutch was included. A search update was carried out in September 2020, but no recent studies were eligible for inclusion. The retrieved results were merged into the citation management software EndNote X9 (Clarivate Analytics, Boston).

### Selection of studies

Studies were screened using a two-step approach: firstly, two authors (AH, HK) independently screened all the abstracts for eligibility. Unclear cases were discussed until consensus was reached. Secondly, the full texts of the included abstracts were assessed for inclusion. In case of disagreement, a third reviewer (VV) was consulted in order to reach consensus. The authors of studies with missing information (e.g., regarding the sample’s age structure) were contacted.

Qualitative studies in which people aged 80 and over (median or average age of study population: at least 80 years) who live at home expressed their views were eligible for inclusion. In addition to studies reporting directly on care preferences, needs and expectations, studies on participants’ positive and negative care experiences were also included, because it was expected that preferences would be derived from these descriptions. Studies on end-of-life care were excluded, since the goals of this can differ strongly from other areas of (geriatric) care. The search and selection criteria are summarized in Table 1.

**Table 1:** Search and selection criteria

	Inclusion	Exclusion
Population	• Participants aged 80 or older • Mean age or median age of study population is 80 or older • Mixed participant groups: inclusion, if results for people aged 80 or older can be separated	• Mean age or median age is under 80 years • Mixed participant groups: exclusion, if results are mixed and cannot be separated for people aged 80 and older
Phenomenon of interest	• Studies on preferences, wishes and needs of older people regarding formal/professional medical or nursing care • Studies on care experiences, problems, determinants and factors of care regarding formal/professional healthcare	• Studies on end of life care, particular therapies • Studies on technical devices and applications • Studies not focusing on healthcare • Studies on informal/unprofessional care or volunteer work
Context/Setting	• Ambulatory/outpatient healthcare (medical and nursing care) • Primary healthcare, general practice • Home healthcare • Participants living at home	• Participants living in an institutional care setting • Hospital care
Study design	Qualitative studies focusing on the perspective and descriptions of older people (interviews, focus groups, group interviews with semi-structured interview guides or open-ended questions)	• Non-qualitative study designs • Studies not focusing on the own perspective and descriptions of older people, e.g., surveys, observations • Mixed-methods designs in which qualitative findings of older people’s perspectives cannot be separated
Language	English, German, Dutch	Other languages
Type of research report	Full research reports	Poster abstracts, editorials, comments, book chapters, study protocols

### Quality appraisal

Two authors (AH, HK) independently evaluated the quality of each included study. Since our aim was to synthesize the qualitative studies’ findings and provide a second-order interpretation, we were especially reliant on their validity, meaning that the findings are reasonable representations of the original data and their contexts, and are convincing and coherent (Leung 2015; Whittemore et al. 2001). This means that data, data collection and analysis had to be appropriate to the respective qualitative research aim (Leung 2015). Therefore, we used the Quality Appraisal Checklist for Qualitative Studies of the National Institute for Health and Care Excellence that examines the appropriateness and coherence of the study instead of item reporting (NICE 2012). Unclear cases were discussed with a third reviewer (VV) where necessary. Quality appraisal was used not to weight individual study contributions, but to evaluate the robustness of the synthesized findings.

### Analysis and synthesis

The results of the included studies formed the basis for the synthesis. In studies reporting on different participant groups, only those parts explicitly referring to the perspective of people aged 80 and over were used. The analysis was conducted using MAXQDA Analytics Pro 2020 (VERBI software, Berlin). In accordance with Thomas and Harden (2008), the analysis consisted of three steps: inductive line-by-line-coding, development of descriptive themes, and development of analytical themes (integration and explanation of the findings).

Firstly, the findings of the primary studies were inductively coded *line-by-line* with regard to their content and meaning. Two authors (AH, HK) independently coded a random sample of four studies. Secondly, both authors categorized them toward an initial set of *descriptive themes* and discussed their results for consensus. Subsequently, the independently examined eight and nine descriptive themes were refined to a set of ten that described relevant aspects of two dimensions: healthcare structures and care relationships. The remaining studies were coded with these themes and in the final consultation, the set was refined to fourteen descriptive themes (Table 2). Moreover, first ideas to explain the themes were collected during this process.

**Table 2:** Characteristics of included studies

Author(s), Year	Title	Country	Data collection and analysis	Sample	Care setting	Care-related background of the older people
Behm et al. (2013)	Preventive home visits and health—experiences among very old people	Sweden	Semi-structured interviews and phenomenographic method	17 participants (80–92 years, 12 female, 7 male) that were considered to be pre-frail and lived at home	Preventive home visit	Pre-frail patients; perceived health was reasonable to excellent, participants were independent from help
Berkelmans et al. (2010)	Characteristics of general practice care: what do senior citizens value? A qualitative study	Netherlands	Semi-structured interviews and framework method	13 patients (65–91 years, mean age 81.2, 7 female, 6 male) from four GP practices;	General practice care	Four patients with rather bad or moderate perceived health, nine patients with reasonable to excellent perceived health
Bjornsdottir (2018)	‘Holding on to life’: an ethnographic study of living well at home in old age	Iceland	Interviews and thematic analysis	15 home care nursing clients (82–99 years, 9 female, 6 male) that were considered to be frail	Nursing home care	Patients that were considered to be frail; participants had several different chronic conditions, health status was rather poor
Faeo et al. (2020)	Home-dwelling persons with dementia’s perception on care support: qualitative study	Norway	Interviews and hermeneutic approach	12 day care center attendants (69–89 years, mean age 82, 6 female, 6 male) diagnosed with dementia and living at home	Care settings with relevance to dementia patients; focus on day care centers	Patients with diagnosis of dementia recruited in day care centers
Gowing et al. (2016)	Patients’ experiences of a multidisciplinary team-led community case management program: a qualitative study	UK	Semi-structured interviews and thematic analysis	16 frail patients (48–90 years, median age 82.5, 11 female, 5 male) and 7 family members	Case management	Frail patients recruited from the Northumberland High Risk Patient Program
Jarling et al. (2018)	Becoming a guest in your own home: home care in Sweden from the perspective of older people with multimorbidity	Sweden	Interviews and content analysis	12 home care clients with multimorbidity (77–90 years, 8 female, 4 male)	Home care	Patients with multimorbidity and several different medical problems; patients received home care and lived alone
King et al. (2018)	Implementation of a gerontology nurse specialist role in primary health care: health professional and older adult perspectives	New Zealand	Semi-structured interviews and content analysis	5 participants who received the intervention (3 female, 2 male (mean age > 80) and 6 healthcare professionals	Case management	Patients at high risk of health and functional decline who received the intervention “primary healthcare gerontology nurse specialist”
Krothe (1992)	Constructions of elderly people's perceived needs for community-based long-term care	USA	Interviews and content analysis	9 clients (65–93 years, mean age 81.4, 7 female, 2 male)	Community-based long-term care	Clients of an Area Agency on Ageing with different conditions and at risk for institutionalization, which needed help with daily activities
Martin-Matthews and Sims-Gould (2008)	Employers, home support workers and elderly clients: identifying key issues in delivery and receipt of home support	Canada	Semi-structured interviews and constant comparative method	14 home care clients (mean age 83, 10 female, 4 male) and 11 home care employers and 32 home support workers	Home care	Clients of home support agencies with different durations of home support (2–250 weeks)
Michel et al. (2015)	From real to ideal—the health (un)care of long-lived elders	Brazil	Interviews and thematic analysis	10 basic health unit users (aged 80 or older, 5 female, 5 male) and 10 nursing professionals	Basic health unit	Older people that were assigned to the basic health unit for at least six months, no further description of health background
Modig et al. (2012)	Frail elderly patients’ experiences of information on medication. A qualitative study	Sweden	Semi-structured interviews and content analysis	12 frail patients (65–88, median age 80.5, 7 female, 5 male)	General, focus on information on medication	Recruitment from a study that evaluated a case manager model; patients taking cardiovascular medications, had been admitted to hospital twice or more and had four or more outpatient care contacts, needed help with at least two activities of daily living
Moe et al. (2013)	The meaning of receiving help from home nursing care	Norway	Narrative interviews and hermeneutic approach	11 home nursing care clients (80–92 years, mean age 88, 6 female, 5 male)	Home care nursing	recruited through a former study on older people; with chronic diseases (e.g., heart disease, diabetes, visual and hearing impairments)
Sandberg et al. (2014)	Case management for frail older people—a qualitative study of receivers’ and providers’ experiences of a complex intervention	Sweden	Open-ended interviews and content analysis	14 older people living at home (75–95 years, mean age 83, 10 female, 4 male) and 6 case managers	Case management in outpatient setting (intervention);	recruited during intervention; participants that needed help with at least two activities of daily living, in the past twelve months two or more admissions to hospital or four contacts to outpatient care
Schulman-Green et al. (2006)	Goal setting as a shared decision-making strategy among clinicians and their older patients	USA	Focus groups and content analysis	42 participants in four focus groups (mean age 81, 25 female, 15 male) and 11 clinicians in two focus groups	General, focus on goal setting in clinical encounter	Participants with at average two chronic conditions and mild to moderate functional impairments, assisted/independent living,
Soodeen et al. (2007)	Home care for older couples: “It feels like a security blanket…”	Canada	Interviews and thematic content analysis	9 home care clients (70–94 years, mean age 80, 6 female, 3 male) and 9 spouses	Home care	Participants with at least one chronic condition
Spoorenberg et al. (2015)	Experiences of community-living older adults receiving integrated care based on the chronic care model: a qualitative study	Netherlands	Semi-structured interviews and grounded theory	23 community-dwelling older people (75–89 years, mean age 82, 13 female, 11 male)	Population-based integrated care model (Embrace)	Care receivers of different health profiles and classifies as either robust, frail or having complex care needs
Tiilikainen et al. (2019)	“They’re always in a hurry”—older people’s perceptions of access and recognition in health and social care services	Finland	Focus groups and thematic analysis	19 participants in four focus groups (mean age 80, 15 female, 4 male)	General, focus on health and social care services	Older people living alone who used health and social care services in the past six months; no further description of health background
Toien et al. (2015)	Older users’ perspectives on the benefits of preventive home visits	Norway	Interviews and hermeneutic approach	10 users of preventive home visits (81–91 years, mean age 85.5, 6 female, 4 male)	Preventive home visits	Two patients had only minor health concerns, the others were physically restricted in varying degrees (mainly neurological and musculoskeletal problems); three were considered to be frail
Turjamaa et al. (2014)	Living longer at home: a qualitative study of older clients’ and practical nurses’ perceptions of home care	Finland	Interviews and content analysis	23 home care clients (mean age 84), and 14 practical nurses	Home care	Clients who received one or two home visits a day
van Blijswijk et al. (2018)	Wishes and needs of community-dwelling older persons concerning general practice: a qualitative study	Netherlands	Group interviews and framework method	24 participants (median age 85.7, 18 female, 6 male)	Integrated care trial	19 participants with multimorbidity and 17 with polypharmacy, several health complaints and physical impairments
van Kempen et al. (2012)	Home visits for frail older people: a qualitative study on the needs and preferences of frail older people and their informal caregivers	Netherlands	Interviews and grounded theory	11 community-dwelling frail participants (65–90, median age 80, 9 female, 2 male), and 11 informal caregivers	Home visits	Most participants with polypharmacy and varying other conditions such as multimorbidity or cognitive/physical impairments,
Walker et al. (2018)	Dementia assessment services: what are the perceptions of older people?	Australia	Semi-structured interviews and content analysis	9 dementia patients (66–90 years, mean age 80, 4 female, 5 male)	dementia assessment services, mainly outpatient specialist services	Patients with a diagnosis of mild dementia

Thirdly, the similarities and differences in the descriptions of all the aspects were compared theme-by-theme in order to gain an understanding of *why* they matter to older people and develop saturated *analytical themes*. Since we were not working with primary data and therefore, could not rely on a concept of saturation based on the emergence of new codes and potentially conducting more interviews, we built on a concept of meaning saturation and the explanatory power of the analytical themes (Hennink et al. 2017; Saunders et al. 2018). This meant that we did not stop analysis at the point of information redundancy but at the point of the best fit between our primary studies’ findings and the analytical themes. Therefore, one author (AH) compared the themes and suggested a set of analytical themes that best integrated and explained them based on the earlier collection of ideas. In the next step, this was discussed in the research team and the analytical themes were refined. The two steps were repeated and after the next revision, the second author checked the results regarding the analytical themes to validate them. After this step, minor revisions regarding the analytical themes’ wording were conducted and a final discussion with the research team took place that confirmed the analytical themes. While the descriptive themes describe general relevant care aspects, the analytical themes were ultimately understood as the underlying wishes of older people that explained why these aspects are relevant, and what matters to them fundamentally regarding ambulatory care.

## Results

### Systematic review and quality appraisal

**Fig. 1:**
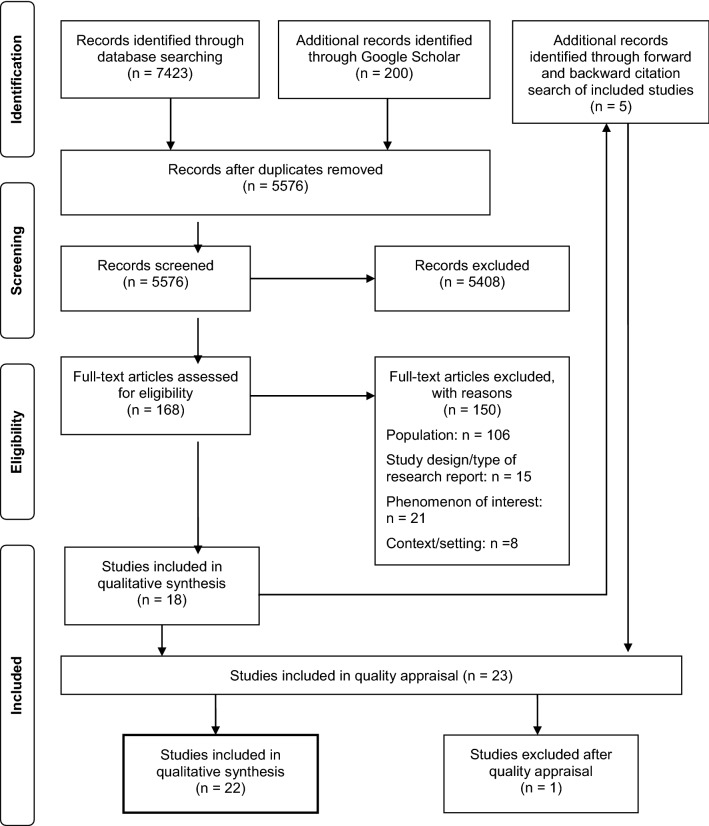
Identification and selection of studies for meta-synthesis based on PRISMA statement

In total, 5576 research reports were identified during the search process. A flowchart for the search and selection process is provided in Fig. 1. Following screening for eligibility, 23 full texts were included for quality appraisal (22 peer-reviewed articles, 1 doctoral thesis). During this step, the article by Krothe (1997) was excluded because her doctoral thesis on the same study sample was also retrieved, and demonstrated higher quality (Online Resource 2). Ultimately, 22 studies were included for meta-synthesis.

### Characteristics of included studies

Most of the included studies were conducted in Northern and Western Europe (*n* = 15), and used interviews for data collection (*n* = 19). The studies comprised 330 eligible older participants in total, approximately two thirds of whom were female. Eight studies assessed the participants’ views regarding home care and community-based long-term care, six studies dealt with ambulatory general practice or specialist care. Moreover, three studies examined (preventive) home visits and case management, respectively. While two studies reported that most of their participants perceived their health status to be reasonable to excellent and another two studies focused on dementia patients, the rest of the included studies reported on older people with several varying chronic conditions, multimorbidity, frailty or at risk for functional decline and institutionalization. Therefore, most of the studies did not examine a specific or acute occasion for seeking care, but the older people’s general experiences with care they received for long-term conditions and related impairments. An overview of the studies’ characteristics is provided in Table 3.

**Table 3:** Explanation of descriptive themes

Descriptive theme	Meaning
*Healthcare structures*	
Time for care	Time that is available for appointments, interactions and care in general
Skills of professionals	Knowledge, technical and communication competencies of healthcare professionals
Sufficient support	Care that is suitable to support the older person with its individual needs
Care coordination	Care that is organized and supervised by a healthcare professional
Access to care	Fast and easy availability of different care services, e.g., specialist care
Continuity and reliability of care	Care that is predictable and provided by familiar persons
Information	Extent, content and manner of information transfer between older person and healthcare professional
Place of care	Regular setting in which care is provided (home/ambulatory versus institutional care)
*Care relationships*	
Involvement in decisions and care	Role and inclusion of the older person in decision processes and care situations
Care contact as social contact	Interactions with care professionals as meaningful social interactions beyond the main reason for care
Friendliness	Attitude and handling of healthcare professionals toward older people
Personal care relationships	Close and trustful relationships between the older person and healthcare professionals
Activation	Motivation and support for the older person to participate in activities
Open and confidential communication	Atmosphere that allows older people to speak uninhibitedly and bring up their problems

### Results of meta-synthesis

**Fig. 2:**
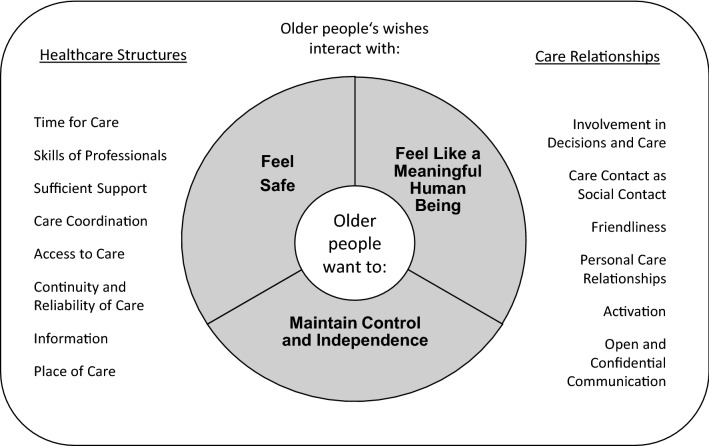
Results of the meta-synthesis. Analytical themes represent underlying wishes (center), descriptive themes represent relevant aspects of healthcare structures (left) and care relationships (right)

We identified three analytical themes as the underlying wishes of older people: (1) *feeling safe*, (2) *feeling like a meaningful human being* and (3) *maintaining control and independence* (Fig. 2). These appear to be of equal importance and do not follow a hierarchy; instead, they rather interact with and complement each other. Despite the second theme that was not present in the studies on case management, the analytical themes represent the diverse range of ambulatory care settings and health conditions of older people as described above. Therefore, the three themes should be understood as set of general underlying wishes of older people regarding ambulatory healthcare structures and care relationships rather than regarding specific treatments or care settings. As the core of the meta-synthesis, the three analytical themes/wishes are described in detail in the following section and complemented by their most significant relations to single care aspects as found in this review.

### Feeling safe

The first fundamental wish was “feeling safe.” Older people had several fears related to their age (e.g., medical emergencies, consequences of polypharmacy, further physical or mental deterioration) that evoked a strong desire for a “safety net” regarding their healthcare and daily life (Behm et al. 2013; Berkelmans et al. 2010; Faeo et al. 2020; Gowing et al. 2016; Jarling et al. 2018; Modig et al. 2012; Moe et al. 2013; Sandberg et al. 2014; Soodeen et al. 2007; Spoorenberg et al. 2015; Toien et al. 2015; Turjamaa et al. 2014; Walker et al. 2018). An 82-year-old male participant in Toien et al. (2015) said:

The most important is the safety—you know, that someone cares and looks after you and checks that the head is still functioning; that is very reassuring. And knowing you are within the municipality’s system (p. 704, preventive home visits).

The strongest contributions to a sense of security among older people were found in aspects of the healthcare structures. Older people felt safe when they received the support they considered necessary, i.e., sufficient, attentive care that met their needs and included individual adjustments (Bjornsdottir 2018; Modig et al. 2012; Soodeen et al. 2007; Toien et al. 2015). The feeling of safety was particularly pronounced when a healthcare professional or case manager monitored their health status and stayed in touch with them (Bjornsdottir 2018; Faeo et al. 2020; Gowing et al. 2016; King et al. 2018; Sandberg et al. 2014; Spoorenberg et al. 2015; Toien et al. 2015; van Blijswijk et al. 2018). Conversely, participants felt insecure when they did not receive the support they needed (Gowing et al. 2016; Modig et al. 2012). In addition to this, continuous, reliable and predictable care was important to the feeling of safety, while participants feared a high turnover of healthcare professionals (Berkelmans et al. 2010; Bjornsdottir 2018; Jarling et al. 2018; Martin-Matthews and Sims-Gould 2008; Modig et al. 2012; Moe et al. 2013; Spoorenberg et al. 2015).

Another important factor for safety was care coordination. Older people felt relieved and safe when their care was coordinated by a healthcare professional or case manager, i.e., when someone organized their care and ensured collaboration between different healthcare providers (Gowing et al. 2016; King et al. 2018; Sandberg et al. 2014; Spoorenberg et al. 2015; Walker et al. 2018). Sandberg et al. (2014) stated:

[…] case management was experienced as something beneficial and something that could contribute to a sense of security (p. 9, case management).

Moreover, being able to access healthcare (e.g., general practice, specialists) and a familiar contact person quickly and easily when necessary was perceived as essential (Behm et al. 2013; Berkelmans et al. 2010; Moe et al. 2013; Spoorenberg et al. 2015; Toien et al. 2015). Likewise, waiting times and not receiving direct access evoked feelings of insecurity (Berkelmans et al. 2010; Modig et al. 2012). The same applied to information, as shown by a statement in Modig et al. (2012):

If the information was limited and there was no one available to answer questions, there were soon feelings of insecurity (p. 6, information on medication).

Receiving sufficient information regarding their care, such as information on medication, was crucial to helping older people feel safe (Modig et al. 2012; Moe et al. 2013). Information on additional services and care options improved the sense of safety (Behm et al. 2013; Turjamaa et al. 2014), as did experience, knowledge and communication skills on the part of healthcare professionals (Berkelmans et al. 2010; Spoorenberg et al. 2015; Walker et al. 2018).

Other factors important to a sense of safety for older people were found in their care relationships. Close and long-term relationships promoted trust and feelings of safety (King et al. 2018; Sandberg et al. 2014; Soodeen et al. 2007). This was closely linked to open communication; if the communication style between the older persons and their professional caregivers was not confidential and trustful, insecurity and distrust increased (Jarling et al. 2018; Modig et al. 2012).

### Feeling like a meaningful human being

This theme represents the external perception and self-perception of aged persons within care relationships. While most of these people had to deal with physical and mental deteriorations such as diminished vision and, consequently, restrictions such as loss of mobility, they focused on the remaining options available to them—they wanted to enjoy their lives despite their old age (Behm et al. 2013; Bjornsdottir 2018; Faeo et al. 2020; Moe et al. 2013; Spoorenberg et al. 2015). The primary studies showed that older people wanted to be seen and accepted as valuable individuals who still act on their own, take pleasure in daily activities and contribute something to society (Behm et al. 2013; Krothe 1992; Martin-Matthews and Sims-Gould 2008; Moe et al. 2013; Tiilikainen et al. 2019; Toien et al. 2015). This was described as being “confirmed […] as a human being” (Moe et al. 2013, p. 744) and “a wish for dignity, value, and self-esteem” (Toien et al. 2015), p. 706). As one 82-year-old woman in the interviews in Toien et al. (2015) put it:

That I am not just sitting here and am forgotten, but that someone makes me feel that I still have something to contribute. That I’m not just a weak human being who sits here, but I still mean something (p. 707, preventive home visits).

However, the studies revealed that older people endured a variety of negative care experiences. Depending on their interaction with their caregivers, they described feeling unimportant, worthless, vulnerable, helpless, overlooked or inferior, and said they were afforded lower priority and interest because of their age (Behm et al. 2013; Bjornsdottir 2018; Jarling et al. 2018; Modig et al. 2012; Moe et al. 2013; Tiilikainen et al. 2019). This was connected to a loss of privacy (in home care) and feeling like a burden to others (Jarling et al. 2018; Moe et al. 2013; Tiilikainen et al. 2019).

In view of this, it was not surprising that whether an older person felt like a meaningful human being was strongly connected to their care relationships. The most important aspect of this was social contact itself, i.e., conversations and interest toward the older people (Behm et al. 2013; Jarling et al. 2018; Krothe 1992; Moe et al. 2013; Soodeen et al. 2007; Tiilikainen et al. 2019). Soodeen et al. (2007) summarized:

[…] becoming acquainted with their [healthcare worker] beyond interacting about the tasks at hand and enjoying a little casual conversation help[ed] put the [care receivers] at ease (p. 1249, home care).

More explicitly, experiencing friendliness and respect made older people feel valuable, while experiencing rudeness and disrespect made them feel burdensome and unimportant (Bjornsdottir 2018; Jarling et al. 2018; Moe et al. 2013; Soodeen et al. 2007; Toien et al. 2015). Such negative care relationship experiences inhibited open communication between the older persons and their healthcare providers (Jarling et al. 2018; Moe et al. 2013). Furthermore, receiving the support they needed promoted a sense of meaningfulness among older people. Individual help enabled them to continue doing things they considered important, and therefore improved their well-being (Moe et al. 2013; Tiilikainen et al. 2019; Toien et al. 2015).

### Maintaining control and independence

The third fundamental wish identified by the older people was to maintain their control and independence. This related to several aspects of their healthcare structures and care relationships. It became apparent that participants adapted to age-related changes on their own, for instance by giving up certain activities, and that they tried to manage as many things as they could on their own (Behm et al. 2013; Bjornsdottir 2018; Krothe 1992; Michel et al. 2015; Modig et al. 2012; Sandberg et al. 2014; Soodeen et al. 2007). A participant in Soodeen et al. (2007) explained:

You’ve got more self-worth, you know, thinking, ‘well, I can do it for myself yet’ (p. 1247, home care).

This illustrates the wish to be independent, and that older people wanted to avoid receiving care and support for as long as possible, because they feared losing control (Behm et al. 2013; Berkelmans et al. 2010; Bjornsdottir 2018; Faeo et al. 2020; Krothe 1992; Sandberg et al. 2014; Spoorenberg et al. 2015; Tiilikainen et al. 2019; van Kempen et al. 2012). Being independent was important to their sense of control and their self-esteem—but they also acknowledged that they needed help to maintain their independence (Behm et al. 2013; Bjornsdottir 2018; Faeo et al. 2020; Gowing et al. 2016; Krothe 1992; Soodeen et al. 2007; Toien et al. 2015).

On the other hand, anecdotes frequently stated that receiving support, such as home care, meant adapting to caregivers’ work routines and schedules, and loss of control, influence and choices, culminating in feelings of dependence and exposure (Gowing et al. 2016; Jarling et al. 2018; Krothe 1992; Moe et al. 2013; Soodeen et al. 2007; Spoorenberg et al. 2015; Tiilikainen et al. 2019; Toien et al. 2015). A female participant in the interviews of Jarling et al. (2018) reported:

I have said, no guys, when I shower… don’t want to show myself when I am old. I feel ashamed. Shame, you’re ashamed… for your body when it becomes old. Those who send me caregivers do not take my privacy into account (p. 4, home care).

It became clear that maintaining control and independence was a delicate balancing act. This proved once more that receiving the support they needed was crucial to older people. Receiving too little or too much support could lead to dependence, whereas the “right” amount of support, i.e., an individually adjusted program, promoted feelings of self-control and independence among older people (Gowing et al. 2016; Krothe 1992; Sandberg et al. 2014). For most older persons, this was only possible at home; institutional care was perceived as a threat to their self-control and independence (Bjornsdottir 2018; Gowing et al. 2016; Jarling et al. 2018; Krothe 1992; Soodeen et al. 2007; Spoorenberg et al. 2015; van Blijswijk et al. 2018). As such, receiving appropriate information on topics such as additional services that would allow them to continue living at home and healthcare professionals who were willing to share their knowledge were important aspects of favorable healthcare (Krothe 1992; Michel et al. 2015; Modig et al. 2012; Toien et al. 2015).

In addition to this, the relationships between healthcare professionals and care receivers affected the older people’s feeling of independence. A close relationship and open, confidential communication were favorable (Jarling et al. 2018; Krothe 1992; Soodeen et al. 2007). Consequently, being involved in decisions and their care helped older people to feel independent and in control, and to achieve their individual goals (Berkelmans et al. 2010; Gowing et al. 2016; Jarling et al. 2018; Krothe 1992; Modig et al. 2012; Moe et al. 2013; Sandberg et al. 2014; Schulman-Green et al. 2006; Spoorenberg et al. 2015; Tiilikainen et al. 2019; Turjamaa et al. 2014; van Blijswijk et al. 2018). Spoorenberg et al. (2015) stated:

The participants made decisions in cooperation with their case managers, which increased their sense of being in control (p. 12, population-based integrated care/case management).

This was complemented by the promotive effects of (physical, mental, social) activation via healthcare professionals (Behm et al. 2013; Krothe 1992; Martin-Matthews and Sims-Gould 2008; Spoorenberg et al. 2015; Toien et al. 2015; van Blijswijk et al. 2018).

## Discussion

The aim of this review was to explore what matters to people aged 80 and over regarding ambulatory care. The meta-synthesis of 22 qualitative studies showed that three underlying wishes shape older people’s perspectives: feeling safe, feeling like a meaningful human being, and maintaining control and independence.

The results are in line with previous meta-studies on the preferences and needs of older people. Dostálová et al. (2020) found six themes in fifteen studies exploring the needs of home care recipients: (1) coping with illness, (2) autonomy, (3) relationship with professionals, (4) quality, safe and secure care, (5) role in society, and (6) environment. The authors stated that in the opinion of older people, good care also counteracts loneliness and includes casual conversations with caregivers, whereas a lack of interest in the care recipients was considered poor-quality care. While Dostálová et al. (2020) focused only on home care, this review shows that the themes are similar for ambulatory medical and nursing care in general. This might be an indication that the results truly represent the fundamental motives of the oldest old, which tend to be related to the general circumstances of their age rather than their specific care dependency. However, consequences of the perception of the older persons as meaningful individuals, by both themselves and others, seem to be more central in our review.

There are also similarities with reviews with a lower average sample age. For example, a qualitative meta-study on the needs of older people in community healthcare stressed the role of maintaining self-esteem and health (Holm et al. 2013). Two central themes were reported: (1) “reconciliation with how life has come” and (2) “desire to regain identity and sense of self-worth despite disability” (p. 6). Autonomy and the older person’s sense of self were also important in studies on healthcare experiences synthesized by Gregory et al. (2017). In line with this research, our work highlights how professional care and support may be both a threat to individual independence and the key factor in the continuation of said independence. This balancing act is a never-ending challenge in older age, although support needs could have been expected to be common and more accepted in this group.

This might be due to a different interpretation of “control” in older age. On this matter, Claassens et al. (2014) conducted a qualitative study to explore the concept of perceived control in healthcare among frail older adults. The authors found that the need for control did not become less important in older age, though it did take a different form. For example, the role of communication and involvement became more important to the perception of control (Claassens et al. 2014). This is in line with our findings on the significance of care relationships and care involvement to the feeling of maintaining control. The concept study also showed healthcare aspects that are able to strengthen older people’s feeling of control that we also identified, such as being monitored, care coordination, and trustful relationships (Claassens et al. 2014).

Overall, our findings are similar to meta-studies on the needs and preferences of people aged 65–80, and do not show substantial differences. However, the underlying wishes that were revealed in the meta-synthesis emphasize the social dimension of care more strongly than it is found in functional care structures. This may be due to the fact that older persons need and use healthcare more frequently, so healthcare becomes a significant part of their daily lives (Marengoni et al. 2011; van den Bussche et al. 2011; WHO 2015b). Our review shows that people aged 80 and over generally consider the incorporation of their emotional and social needs during care interactions to be integral to favorable ambulatory care. For those affected by social isolation, these aspects become even more important (Nicholson 2012).

By contrast, care models applied to older people focus mainly on assessment, care coordination and interdisciplinary treatments. Popular examples include the Chronic Care Model (CCM) and the Patient-Centered Medical Home, which are often used as basis for care interventions (Bodenheimer et al. 2002; John et al. 2020). Attempts to adopt the CCM for geriatric care, such as the Geriatric Care Model, take into account more comprehensive assessments and care coordination, which are designed specifically to cater to older people’s wish to feel safe (Hoogendijk et al. 2016; Muntinga et al. 2012; Muntinga et al. 2015). However, the importance of personal care relationships and strengthening the older person as a meaningful human being do not seem to be represented sufficiently thus far and should be emphasized more strongly. Our results show that casual conversations, genuine interest in the older person, friendliness and respect promote these goals. As such, referring this demographic to other services such as social welfare, as is often proposed in existing models of care, cannot be seen as a complete solution. Instead, it could be worthwhile to focus on the health professionals’ behavior and attitude toward older people and adapt care structures accordingly (e.g., by raising awareness of social needs and providing more time for care).

### Strengths and limitations

To our knowledge, this is the first qualitative meta-synthesis on ambulatory healthcare needs and preferences from the genuine perspective of people aged 80 and over. The chosen search strategy enabled the consideration of a comprehensive research status, and the systematic analysis approach ensured intersubjectively valid, i.e., trustworthy and coherent results. Although the 22 included studies focused on different research questions and aspects of ambulatory healthcare, the results are mostly unambiguous, the core of the final three analytical themes emerged fast in the analysis process and further steps mainly addressed their wording and clarifications of their understanding. Therefore, we assume that the analytical themes provide a reasonable integration and explanation of the primary studies’ findings and can be considered saturated in their meaning. Furthermore, the methodological quality of the individual studies was found to be sufficient according to the quality appraisal specifically encompassing trustworthiness, coherence, and the appropriateness of the research design; this strengthens the validity of the results.

However, several limitations must be considered. Firstly, there is a possibility of dissemination bias if qualitative studies or parts of their results are not made available in full (Booth et al. 2018). The study sample is also limited by the exclusion of languages other than English, German and Dutch. Additionally, the average age of potentially eligible studies’ samples was often unclear, and some authors did not respond to our requests for contact; this resulted in the exclusion of the studies in question. Despite the use of a comprehensive research strategy including an update after one year, further or contradictory research results may not have been considered.

Secondly, the findings are only applicable to developed and high-income countries, since the included studies were conducted in such countries. Primarily due to the lower average sample age, studies from low-income countries had to be excluded during screening. Since there are indications that accessibility and affordability of care are far more important issues for older people in these countries and preferences may differ depending on cultural background and known care structures, caution should be exercised if transferring the results (WHO 2015b). Further studies are needed in the countries not covered by this review, though our results could serve as a basis for their design and analysis.

Thirdly, the studies included do not represent the full range of (medical and nursing) care and services necessary to age in place. Moreover, the evidence from qualitative studies presented in this review hardly covers acute occasions for seeking ambulatory care (e.g., acute exacerbations of a chronic condition) and it is possible that older people’s priorities and preferences are different in these care situations. In order to design comprehensive older-people-centered care, the perspective of people aged 80 and over should be researched further with regard to acute care (also in combination with chronic care) and specialties such as pharmacy and dental care.

### Conclusion

This review highlights the fundamental wishes that matter to older people regarding ambulatory healthcare: feeling safe, feeling like a meaningful human being and maintaining control and independence. They interact with several aspects of ambulatory healthcare structures and care relationships that were identified as relevant. In order to achieve patient-centered care for the oldest old, future care models and policies should be developed and evaluated based on these wishes. Furthermore, the relationship between the (fulfillment of) identified wishes on patient-reported experiences and outcomes, such as well-being and satisfaction with care, should be investigated further in order to gain a better understanding of ambulatory care favored by older people.

## Supplementary Information

Online Resource 1: Search strategy for PubMed.

Online Resource 2: Results of quality appraisal per study.

Below is the link to the electronic supplementary material.

Supplementary file1 (PDF 66 kb)

Supplementary file2 (PDF 56 kb)

## Data Availability

All data and material is available from the authors on request.
