# Supplementary material for: What matters to people aged 80 and over regarding ambulatory care? A systematic review and meta-synthesis of qualitative studies
Source: Eur J Ageing. 2021 Aug 21;19(3):325–39. doi: 10.1007/s10433-021-00633-7 (PMC9424416; doi:10.1007/s10433-021-00633-7)
Supplement: Supplementary file 1 — Supplementary file1 (PDF 66 kb) [file 10433_2021_633_MOESM1_ESM.pdf]

Herrler A, Kukla H, Vennedey V, Stock S. What matters to people aged 80 and over regarding ambulatory care? A systematic review and meta-synthesis of qualitative studies. European Journal of Ageing.

Corresponding author: Angélique Herrler, Faculty of Human Sciences and Faculty of Medicine, Graduate School GROW – Gerontological Research on Well-being, University of Cologne, Albertus-Magnus-Platz, 50923 Cologne, Germany; e-mail: angelique.herrler@uni-koeln.de

Online Resource 1: Search strategy for PubMed

((((((((((((((((((((aged, 80 and over[MeSH Major Topic]))) OR frail elderly[MeSH Major Topic]) OR geriatric[Title/Abstract]) OR ((octogenarian[Title/Abstract] OR octogenarians[Title/Abstract]))) OR ((nonagenarian[Title/Abstract] OR nonagenarians[Title/Abstract]))) OR ((centenarian[Title/Abstract] OR centenarians[Title/Abstract]))) OR oldest[Title/Abstract]) OR ((elderly[Title/Abstract] OR elder[Title/Abstract] OR elders[Title/Abstract] OR eldest[Title/Abstract]))) OR "advanced age"[Title/Abstract]) OR "late age"[Title/Abstract]) OR "old age"[Title/Abstract])) AND (((((patient-centered care[MeSH Major Topic]) OR ((offer[Title/Abstract] OR offers[Title/Abstract]))) OR healthcare[Title/Abstract]) OR ((service[Title/Abstract] OR services[Title/Abstract]))) OR care[Title/Abstract]))) OR health services for the aged[MeSH Terms])) AND ((((((((((((((((\"day care"[Title/Abstract]) OR adult day care centers[MeSH Terms]) OR "family practice"[Title/Abstract]) OR family practice[MeSH Terms]) OR "general practice"[Title/Abstract]) OR general practice[MeSH Terms]) OR ((home[Title/Abstract] OR homebound[Title/Abstract]))) OR home care services[MeSH Terms]) OR ((community[Title/Abstract] OR communities[Title/Abstract] OR community-dwelling[Title/Abstract]))) OR community health services[MeSH Terms]) OR primary[Title/Abstract]) OR primary health care[MeSH Terms]) OR outpatient[Title/Abstract]) OR ((ambulatory[Title/Abstract] OR ambulant[Title/Abstract]))) OR ambulatory care[MeSH Terms]) OR ambulatory care facilities[MeSH Terms])) AND (((((((((((("grounded theory"[Title/Abstract]) OR grounded theory[MeSH Terms]) OR (("focus group"[Title/Abstract] OR focusgroup[Title/Abstract] OR "focus groups"[Title/Abstract] OR focusgroups[Title/Abstract]))) OR focus groups[MeSH Terms]) OR ((interview[Title/Abstract] OR interviews[Title/Abstract]))) OR interviews as topic[MeSH Terms]) OR "qualitative study"[Title/Abstract]) OR "qualitative analysis"[Title/Abstract]) OR "qualitative evaluation"[Title/Abstract]) OR "qualitative research"[Title/Abstract]) OR qualitative research[MeSH Terms])) OR ((((phenomenologic[Title/Abstract] OR phenomenological[Title/Abstract]))) OR ((in-depth[Title/Abstract] OR indepth[Title/Abstract]))))) AND ((((((((((((((((factor[Title] OR factors[Title]))) OR ((determinant[Title/Abstract] OR determinants[Title/Abstract]))) OR ((difficulty[Title/Abstract] OR difficulties[Title/Abstract]))) OR ((challenge[Title/Abstract] OR challenges[Title/Abstract]))) OR ((problem[Title/Abstract] OR problems[Title/Abstract]))) OR ((obstacle[Title/Abstract] OR obstacles[Title/Abstract]))) OR ((experience[Title/Abstract] OR experiences[Title/Abstract]))) OR demand[Title]) OR ((need[Title] OR needs[Title]))) OR ((health services needs and demand[MeSH Terms]))) OR ((expectation[Title/Abstract] OR expectations[Title/Abstract]))) OR ((wish[Title/Abstract] OR wishes[Title/Abstract]))) OR ((preference[Title/Abstract] OR preferences[Title/Abstract]))) OR patient preferences[MeSH Terms])
