# Supplementary material for: What matters to people aged 80 and over regarding ambulatory care? A systematic review and meta-synthesis of qualitative studies
Source: Eur J Ageing. 2021 Aug 21;19(3):325–39. doi: 10.1007/s10433-021-00633-7 (PMC9424416; doi:10.1007/s10433-021-00633-7)
Supplement: Supplementary file 2 — Supplementary file2 (PDF 56 kb) [file 10433_2021_633_MOESM2_ESM.pdf]

Herrler A, Kukla H, Vennedey V, Stock S. What matters to people aged 80 and over regarding ambulatory care? A systematic review and meta-synthesis of qualitative studies. European Journal of Ageing.
Corresponding author: Angélique Herrler, Faculty of Human Sciences and Faculty of Medicine, Graduate School GROW – Gerontological Research on Well-being, University of Cologne, Albertus-Magnus-Platz, 50923 Cologne, Germany; e-mail: angelique.herrler@uni-koeln.de

Online Resource 2: Results of Quality Appraisal per Study

Applied Quality Appraisal Tool: NICE (2012) Quality Appraisal Checklist - Qualitative Studies. In: Methods for the development of NICE public health guidance (third edition). National Institute for Health and Care Excellence London.

	Behm, L., S. D. Ivanoff and L. Ziden (2013). "Preventive home visits and health--experiences among very old people."	Berkelmans, P. G., A. J. Berendsen, P. F. Verhaak and K. van der Meer (2010). "Characteristics of general practice care: what do senior citizens value? A qualitative study."	Bjornsdottir, K. (2018). "'Holding on to life': An ethnographic study of living well at home in old age."	Faeo, S. E., F. K. Bruvik, O. Tranvag and B. S. Husebo "Home-dwelling persons with dementia's perception on care support: Qualitative study."	Gowing, A., C. Dickinson, T. Gorman, L. Robinson and R. Duncan (2016). "Patients' experiences of a multidisciplinary team-led community case management programme: a qualitative study."	Jarling, A., I. Rydstrom, M. Ernsth-Bravell, M. Nystrom and A. C. Dalheim-Englund (2018). "Becoming a guest in your own home: Home care in Sweden from the perspective of older people with multimorbidities."	King, A. I. I., M. L. Boyd, L. Dagley and D. L. Raphael (2018). "Implementation of a gerontology nurse specialist role in primary health care: Health professional and older adult perspectives."	Krothe, J. S. (1992). Constructions of elderly people's perceived needs for community-based long-term care.	Krothe, J. S. (1997). "Giving voice to elderly people: community-based long-term care."	Martin-Matthews, A. and J. Sims-Gould (2008). "Employers, home support workers and elderly clients: identifying key issues in delivery and receipt of home support."	Michel, T., M. Helena Lenardt, M. Hautsch Willig and A. Maria Alvarez (2015). "From real to ideal - the health (un)care of long-lived elders."
**Theoretical Approach**											
**1. Is a qualitative approach appropriate?**	Appropriate	Appropriate	Appropriate	Appropriate	Appropriate	Appropriate	Appropriate	Appropriate	Appropriate	Appropriate	Appropriate
Reference	p2/3-4	p2/3	p1/1	p3/1	p2/5	p3/2	p809/2	p6	p218/4	p70/6	p344/4-5
Comments	description of experiences	subjective preferences regarding GP care	understanding how frail older people manage to live at home	description of perceptions and experiences	exploration of experiences	exploration of experiences and meaning	exploration of views regariding intervention	identification of subjective needs	understanding context for continuing to live at home	perspectives of older people regarding delivery of services	meaning of healthcare for older people and nursing professionals
**2. Is the study clear in what it seeks to do?**	Clear	Clear	Clear	Clear	Clear	Clear	Clear	Clear	Clear	Clear	Clear
Reference	p1/1, p2/3-4	p2/3	p1/1, p2/3	p2/4, p3/1	p2/5	p3/2	p809/2	p6	p218/4	p70/6	p344/4-5
Comments	experiences and consequences of a home visit	subjective preferences regarding GP care	understanding how frail older people manage to live at home	description of perceptions and experiences	exploration of experiences	exploration of experiences and meaning	exploration of views regariding intervention	identification of subjective needs	understanding context for continuing to live at home	perspectives of older people regarding delivery of services	meaning of healthcare for older people and nursing professionals
**Study design**											
**3. How defensible/rigorous is the research design/methodology?**	Defensible	Defensible	Defensible	Defensible	Defensible	Defensible	Defensible	Defensible	Defensible	Defensible	Defensible
Reference	p2/7	p2/7	p2/6	p3/4	p2/5-7	p3/3-5	p809/3	p34, p38	p218/5-6	p70/7-8, p711-4	p344/8-10
Comments	purposeful selection, phenomenographic approach	judgement sample; qualitative explorative investigation; research paradigm not clear, probably phenomenology	ethnography; sampling strategy not described, but access - probably convenience sample	hermeneutic; sampling strategy not described, only inclusion criteria	qualitative explorative investigation; research paradigm not clear, probably phenomenology; convenience sample	lifeworld approach, describing a phenomenon, purposive sample	qualitative descriptive design; research paradigm not clear, probably phenomenology, purposive sample	constructivist paradigm; naturalistic methodology, inquiry; purposive sample	constructivist paradigm; inquiry; purposive sample	explorative interviews (phenomenology), purposive sample	qualitative, ethnographic survey; sampling strategy not described
**Data Collection**											
**4. How well was the data collection carried out?**	Appropriately	Appropriately	Appropriately	Appropriately	Appropriately	Appropriately	Appropriately	Appropriately	Not sure/Inadequately reported	Appropriately	Appropriately
Reference	p2/7	p3/1	p3/1	p3/4	p2/9-10	p3/4-5	p809/1-2	p43-45	p218/9-10	p70/7	p344/9-10
Comments	semi-structured interviews	semi-structured interviews, questionnaire	oberservations and interviews	open interview guide	semi-structured interviews	open interviews	semi-structured interviews	unstructured interviews	interviews; structure and setting unclear	semi-structured interviews	semi-structured interviews and participatory observations
**Trustworthiness**											
**5. Is the role of the researcher clearly described?**	Unclear	Clearly described	Unclear	Unclear	Clearly described	Unclear	Unclear	Clearly described	Not described	Not described	Unclear
Reference	p2/6	p3/1-2	p3/1	p3/4	p2/9, supplemental material	p3/4-5	p807	p36, p40			p344/9
Comments	"small talk" and information on procedure; no more details	description of background and study process	frequency of contact, only one researcher; what people knew about her and her background remains unclear	only little details (researchers had clinical experience); relationship and influence of researchers' backgrounds remain unclear	role and independency from intervention provider described; research aim described	contact person insufficiently described as well as interviewer	Degree/job on title page, but no more description; also no reflection in discussion	reflection on role as inquirer described	no description on background or reflection	no description on background or reflection	field work is mentioned, but it is unclear whether and what kind of relationship was established; no further description of researcher or background
**6. Is the context clearly described?**	Clear	Clear	Clear	Clear	Clear	Clear	Unclear	Clear	Clear	Clear	Clear
Reference	p2/7, table 1	p2/4, table 1	p2/7, p3/5, table S1	p3/3, p4/1	p2/7-9	p3/4-5	p810/2	p43	p218/10, table 1	p70/6	p344/5-7
Comments	home environments, different living conditions	diverse characteristics, GP setting	home care agency, home environment	day care centers (and home environment)	home environment, special care programme, different living situations	home environment	home environment, home care; quite generic, more details would be helpful	home environment, long-term care at home	community-based long-term care	community-based care	basic health unit, older people
**7. Were the methods reliable?**	Reliable	Reliable	Reliable	Reliable	Reliable	Reliable	Reliable	Reliable	Not sure	Reliable	Reliable
Reference	p2/7	p2/3, p2/7	p2/6, p3/1	p3/4	p2/9	p3/3	p809/3	p34, p44, p52-53	p218-219	p70/6	p344/9
Comments	only one method, but no necessary indication for more than one method	interviews & quantitative assessment for health status	interviews & observations	only one method, but no necessary indication for more than one method	only one method, but no necessary indication for more than one method	only one method, but no necessary indication for more than one method	quantitative and qualitative, quantitative part described elsewhere	descriptions and perceptions in journal after every interview	description of interview and analysis is too short, no real insight	only one method, but no necessary indication for more than one method	interviews and observations
**Analysis**											
**8. Is the data analysis sufficiently rigorous?**	Rigorous	Rigorous	Rigorous	Rigorous	Rigorous	Rigorous	Rigorous	Rigorous	Not sure/not reported	Rigorous	Rigorous
Reference	p3/3, p4/1-2	p3/4-5	p3/3	p4/2	p3/1-2	p3/7	p810/3	p43-51	p218	p71/5, p72/1	p344/11-12
Comments	phenomenographic method, systematic process	thematic content analysis, systematic process	case studies and thematic analysis, systematic process	hermeneutic analysis, systematic process	thematic analysis, systematic process	qualitative content analysis, systematic process	thematic analysis, systematic process	constant comparison method, systematic process	no description of analysis	constant comparative method, systematic approach	taxonomic analysis, systematic process
**9. Is the data 'rich'?**	Rich	Rich	Rich	Rich	Rich	Rich	Not sure/not reported	Rich	Not sure/not reported	Rich	Rich
Reference	p4/4-6, table 3	p3-5	p3-5	p4-7	p3-5	p4-6	p810-812	p155-196	p219-223	p72-74	p344-348
Comments	comparisons and description of deviating category	comparisons within categories, relation to patient characteristics	good insight in living circumstances and description of single cases, but no comparison regarding differences; rather description of similarities	description of results is connected to individual cases; but no explicit description of differences	detailed description of experiences and limitations of home care	detailed description of experiences, but no real comparison or presentation of diverse cases and differences	detailed description, but only for positive evaluation; no real comparison or presentation of diverse cases and differences; no deviant findings for the perspective of older people	very detailed description of cases and themes	not mentioned, which minor topics appeared; no systematic comparison of, e.g., people with nursing home experiences and people without	detailed descriptions of relevant issues, positive as well as negative aspects described	descriptions are sufficient, but explanations of the investigated phenomena remain short
**10. Is the analysis reliable?**	Reliable	Reliable	Not sure/Not reported	Reliable	Reliable	Not sure/Not reported	Reliable	Reliable	Not sure/Not reported	Reliable	Not sure/Not reported
Reference	p4/3	p3/2-3	p3/4	p4/2	p3/1-2	p3/7	p810/3	p43, p48	p218	p71/5, p72/1	p344/11-12
Comments	more than one researcher, third for validation	two researchers, discussion	only one researcher, but with constant subjective reflection	two researchers, discussion	several researchers with diverse backgrounds, discussion	unclear involvement of research team and where more than one researcher was involved	several researchers including independent researcher	only one researcher, but member checking	no description of analysis; member checking involved	several meetings and discussion of chosen sample	it remains unclear who conducted the analysis
**11. Are the findings convincing?**	Convincing	Convincing	Convincing	Convincing	Convincing	Convincing	Convincing	Convincing	Convincing	Convincing	Convincing
Reference	p4/4-7, p5-6	p3-5	p3-5	p4-7	p3-5	p4-6	p810-812	p155-196	p219-223	p72-74	p344-348
Comments	appropriate referencing and coherent presentation of original citations	appropriate referencing and coherent presentation of original citations	appropriate referencing and coherent presentation of original citations	appropriate referencing and coherent presentation of original citations	appropriate referencing and coherent presentation of original citations	appropriate referencing and coherent presentation of original citations	appropriate referencing and coherent presentation of original citations	appropriate referencing and coherent presentation of original citations	appropriate referencing and coherent presentation of original citations	appropriate referencing and coherent presentation of original citations	appropriate referencing and coherent presentation of original citations
**12. Are the findings relevant to the aims of the study?**	Relevant	Relevant	Relevant	Relevant	Relevant	Relevant	Relevant	Relevant	Relevant	Relevant	Relevant
Reference	p9/3-4	p6-7	p6/6	p9/5-p10/1	p5/18-p6/6	p8/3	p816/5	p211-212	p225	p74	p348/15, p349
Comments											
**13. Conclusions**	Adequate	Adequate	Adequate	Adequate	Adequate	Adequate	Adequate	Adequate	Adequate	Adequate	Adequate
Reference	p9/3-4	p7/11-12	p6/6	p9/5-p10/1	p5/18-p6/6	p8/3	p816/5	p211-212	p225	p74	p348/15, p349
Comments	plausible and appropriate answer to research question	plausible and appropriate answer to research question	plausible and appropriate answer to research question	plausible and appropriate answer to research question	plausible and appropriate answer to research question	plausible and appropriate answer to research question	plausible and appropriate answer to research question; lacking deviant findings may be suspicious	plausible and appropriate answer to research question	plausible and appropriate answer to research question	plausible and appropriate answer to research question	plausible and appropriate answer to research question
**Ethics**											
**14. How clear and coherent is the reporting of ethics?**	Appropriate	Not sure/Not reported	Appropriate	Appropriate	Appropriate	Appropriate	Appropriate	Appropriate	Not sure/Not reported	Not sure/Not reported	Appropriate
Reference	p2/8; p3/3	p2/4, p3/1	p3/2	p3/3	p6	p3/8-p9/1	p809/5	p40			p344/13
Comments	basics available (ethics approval), could be more detailed	ethical approval not required due to national regulations	ethical approval, informed consent, protection of identity	basics available (ethics approval), could be more detailed	basics available (ethics approval), could be more detailed	ethical approval and description of information that participants received	ethical approval and description of information that participants received	ethical approval and description of information that participants received	ethical approval not mentioned	ethical approval not mentioned	ethical approval and description of information that participants received
**Overall assessment**											
**As far as can be ascertained from the paper, how well was the study conducted? (see guidance notes)**	++	++	++	++	++	++	++	++	+	++	++
Comments											

Modig, S., J. Kristensson, M. Troein, A. Brorsson and P. Midlöv (2012). "Frail elderly patients' experiences of information on medication. A qualitative study."	Moe, A., O. Hellzen and I. Enmarker (2013). "The meaning of receiving help from home nursing care."	Sandberg, M., U. Jakobsson, P. Midlov and J. Kristensson (2014). "Case management for frail older people - a qualitative study of receivers' and providers' experiences of a complex intervention."	Schulman-Green, D. J., A. D. Naik, E. H. Bradley, R. McCorkle and S. T. Bogardus (2006). "Goal setting as a shared decision making strategy among clinicians and their older patients."	Soodeen, R. A., D. Gregory and J. B. Bond (2007). "Home care for older couples: "It feels like a security blanket.. ":"	Spoorenberg, S. L. W., K. Wynia, A. S. Fokkens, K. Slotman, H. P. H. Kremer and S. A. Reijneveld (2015). "Experiences of Community-Living Older Adults Receiving Integrated Care Based on the Chronic Care Model: A Qualitative Study."	Tiilikainen, E., A. Hujala, S. Kannasoja, S. Rissanen and K. Närhi (2019). ""They're always in a hurry" – Older people´s perceptions of access and recognition in health and social care services."	Toien, M., I. T. Bjork and L. Fagerstrom (2015). "Older users' perspectives on the benefits of preventive home visits."	Turjamaa, R., S. Hartikainen, M. Kangasniemi and A. M. Pietila (2014). "Living longer at home: a qualitative study of older clients' and practical nurses' perceptions of home care."	van Blijswijk, S. C. E., C. S. de Waard, P. G. van Peet, D. Keizer, M. von Faber, M. W. M. de Waal, W. P. J. den Elzen, J. Gussekloo and J. W. Blom (2018). "Wishes and needs of community-dwelling older persons concerning general practice: A qualitative study."	van Kempen, J. A., S. H. Robben, S. U. Zuidema, M. G. Rikkert, R. J. Melis and H. J. Schers (2012). "Home visits for frail older people: a qualitative study on the needs and preferences of frail older people and their informal caregivers."	Walker, R., J. Ratcliffe, A. White and R. Visvanathan (2018). "Dementia assessment services: What are the perceptions of older people?"
Appropriate	Appropriate	Appropriate	Appropriate	Appropriate	Appropriate	Appropriate	Appropriate	Appropriate	Appropriate	Appropriate	Appropriate
p2/1-2	p738/2	p2/3-4	p145/2, p146/1	p1246/3	p2/3-p3/2	p1012/4	p701/2-3	p3208/1-2	p2/2-3	p555/2-3	p43/3, p44/1
experiences regarding receiving information	meaning of receiving help from home nursing care	experiences after an intervention	explanation of shared decision making preferences	description of home care experiences	opinions and experiences regariding CCM-based integrated care	exploration of connection between health/social care and quality of life	experiences with preventive home visits	description of home care structures and exploration of enabling factors	experiences and expectations regarding GP care	preferences regarding home visits	perceptions of facilitators and barriers in dementia diagnostic services
Clear	Clear	Clear	Clear	Clear	Clear	Clear	Clear	Clear	Clear	Clear	Clear
p2/1-2	p738/2	p2/3-4	p145/2, p146/1	p1246/3	p2/3-p3/2	p1012/4	p701/2-3	p3208/1-2	p2/2-3	p555/2-3	p43/3, p44/1
experiences regarding receiving information	meaning of receiving help from home nursing care	experiences after an intervention	explanation of shared decision making preferences	description of home care experiences	opinions and experiences regariding CCM-based integrated care	exploration of connection between health/social care and quality of life	experiences with preventive home visits	description of home care structures and exploration of enabling factors	experiences and expectations regarding GP care	preferences regarding home visits	perceptions of facilitators and barriers in dementia diagnostic services
Defensible	Defensible	Defensible	Defensible	Defensible	Defensible	Defensible	Defensible	Defensible	Defensible	Defensible	Defensible
p2/3	p738/2-4	p2/5-6, p3/1	p146/2-4	p1246/4-6	p4/1-4	p1012/5-9, p1013/1-4	p702/3-8, p703/1	p3208/3-4	p2/4-5, p3/1-3	p555/4-6	p44/2-4
explorative interviews (phenomenology)	narrative interviews, life world interview	explorative interviews (phenomenology); inclusion criteria described; purposeful sampling strategy for interview pool (not for participants)	qualitative focusgroups, purposive sampling	explorative interviews (phenomenology), convenience sample	grounded theory approach, maximum variation sampling	explorative focus groups, inclusion criteria described but not the sampling strategy	explorative design with hermeneutical approach, purposive sample/maximum variation sampling strategy	descriptive qualitative design, no sampling strategy described	focus groups and indidividual interviews; framework method (content analysis); no sampling strategy described	semi-sctructured interviews, purposive sampling	interpretive phenomenology, purposive sampling
Appropriately	Appropriately	Appropriately	Appropriately	Appropriately	Appropriately	Appropriately	Appropriately	Appropriately	Appropriately	Appropriately	Appropriately
p3/1	p738/5	p2/5-6, p3/1, p4/4-5	p146/5-6, table 1	p1246/7-p1247/1	p4/1-2, p51-2	p1012/5	p703/6-7	p3208/5--3209/3	p3/2-4	p555/6	p44/4
semi-structured interviews	narrative interviews	open-ended interviews, thorough description	focus groups with semi-structured interview guides	explorative interviews	interviews	focus groups	semi-structured interviews	interviews, stimulated by videotaped home visits	semi-structured focus groups	semi-structured interviews	in-depth interviews
Clearly described	Not described	Not described	Not described	Not described	Unclear	Not described	Unclear	Unclear	Not described	Unclear	Not described
p3/1					p4/2-3		p712	p3206, p3209/3		p555/4, p555/6	
researcher is a GP with experience, but was not involved in medical care of interview participants	no description of background or reflection of role	no description of background or reflection of role	no description of background or reflection of role	no description of background or reflection of role	research aim was known; role of researcher unclear; only mentioned that they were trained	no description of background or reflection of role	degrees/background mentioned, but no reflection of role or relationship to participants	degrees/background mentioned, but no reflection of role or relationship to participants	no description of background or reflection of role	degrees/background mentioned, but no reflection of role or relationship to participants	no description of background or reflection of role
Clear	Clear	Clear	Clear	Clear	Clear	Clear	Clear	Clear	Clear	Clear	Clear
p2/1-2, p3/1	p738/2	p2/6, p3/1, p4/2	p146/2	p1246/4	p3	p1012/4	p701/2-3	p3208/4	p2/5, p3/1	p555/4, p555/3	p44/1, p44/4
information on medication, home environment	home nursing care	case management, home environment	independent/assistive living (not institutional)	home care, older married couples	CCM-based integrated care, home enrionment, Embrace model	finnish health and social care services and elderly care	preventive home visits, home environment, p702/4	home care	GP care	frail older adults and informal caregivers, home visits	dementia patients and family carers, dementia diaagnostic services
Reliable	Reliable	Reliable	Reliable	Reliable	Reliable	Reliable	Reliable	Reliable	Reliable	Reliable	Reliable
p3/1	p738/5	p2/6, p3/1	p146/2-6	p1246/4-p1247/1	p4/5, table 1, p5/1-2	p1012/5-8	p703/6-7	p3208/5--3209/3	p3/2-4	p555/4-6	p44/4
only one method, but no necessary indication for more than one method	only one method, but no necessary indication for more than one method	only one method, but no necessary indication for more than one method	only one method, but no necessary indication for more than one method	only one method, but no necessary indication for more than one method	qualitative interviews and quantitative assessment for background characteristics, e.g., Embrace profile	only one method, but no necessary indication for more than one method	only one method, but no necessary indication for more than one method	interviews, stimulated by videotaped home visits	field notes were made during interviews and discussed afterwards	only one method, but no necessary indication for more than one method	only one method, but no necessary indication for more than one method
Rigorous	Rigorous	Rigorous	Rigorous	Rigorous	Rigorous	Rigorous	Rigorous	Rigorous	Rigorous	Not sure/not reported	Rigorous
p3/2	p738/7-p739	p4/6, p5/1	p146/5-6	p1247/2	p5/3, p7/1-5	p1012/9-p1013/4	p703/8-, p704/1-2	p3209/4	p3/5	p555/7	p44/5-6
qualitative content analysis, systematic process	hermeneutic/phenomenological interpretation, systematic process	content analysis, systematic process	content analysis, systematic process	thematic content analysis, systematic process	grounded theory approach, systematic process	thematic analysis, systematic process	hermeneutic approach, systematic process	inductive content analysis, systematic process	qualitative content analysis, systematic process	grounded theory approach, description is too short/details rather unclear	inductive content analysis, constant comparison; systematic process
Rich	Rich	Rich	Rich	Rich	Rich	Rich	Rich	Rich	Rich	Rich	Rich
p3-7	p740-744	p5-10	p146-p149	p1247-1252	p7-12	p1012-1016	p704-707	p3209-3213	p3/6-p10	p556-557	p44-46
detailed description of relevant issues, explanation of relations, positive as well as negative issues displayed	detailed description of relevant issues, explanation of relations, positive as well as negative issues displayed	detailed description of relevant issues, explanation of relations, positive as well as negative issues displayed	detailed description of relevant issues, explanation of relations, positive as well as negative issues displayed	detailed description of relevant issues, explanation of relations, positive as well as negative issues displayed	detailed description of relevant issues, explanation of relations, positive as well as negative issues displayed	detailed description of relevant issues, explanation of relations, positive as well as negative issues displayed	detailed description of relevant issues, explanation of relations, positive as well as negative issues displayed	detailed description of relevant issues, explanation of relations, positive as well as negative issues displayed	detailed description of relevant issues, explanation of relations, positive as well as negative issues displayed	short but sufficient description of relevant topics, positive as well as negative issues explained	detailed description of relevant issues, explanation of relations, positive as well as negative issues displayed
Reliable	Reliable	Reliable	Reliable	Not sure/Not reported	Reliable	Not sure/Not reported	Reliable	Reliable	Reliable	Reliable	Reliable
p3/2	p739	p4/6, p5/1	p146/5-6	p1247/2	p5/3, p7/1-5	p1012/9-p1013/4	p703/8-, p704/1-2	p3209/4	p3/5	p555/7	p44/5-6
main analysis done by first author, validation and discussion with other authors	thorough description, all authors involved	test sample and validation by second researcher, joint discussion	three researchers indepently identified themes in test sample & furhter transcripts, joint discussions	it remains unclear who conducted the analysis	code development by two interviewers, validated by author	it remains unclear who conducted the analysis	all authors involved in coding and categorization	first stage by one researcher, later joint analysis	at least two involved in coding, joint discussion	two independently coding researchers, joint discusssion	independent coding of test sample by two researchers, joint discussion
Convincing	Convincing	Convincing	Convincing	Convincing	Convincing	Convincing	Convincing	Convincing	Convincing	Convincing	Convincing
p3-7	p740-744	p5-10	p146-p149	p1247-1252	p7-12	p1012-1016	p704-707	p3209-3213	p3/6-p10	p556-557	p44-46
appropriate referencing and coherent presentation of original citations	appropriate referencing and coherent presentation of original citations	appropriate referencing and coherent presentation of original citations	coherent presentation of original citations, but no referencing to interview number	coherent presentation of original citations, but no referencing to interview number	appropriate referencing and coherent presentation of original citations	appropriate referencing and coherent presentation of original citations	appropriate referencing and coherent presentation of original citations	appropriate referencing and coherent presentation of original citations	coherent presentation of original citations, but no referencing to interview number	appropriate referencing and coherent presentation of original citations	appropriate referencing and coherent presentation of original citations
Relevant	Relevant	Relevant	Relevant	Relevant	Relevant	Relevant	Relevant	Relevant	Relevant	Relevant	Relevant
p9/3	p745/5-746	p11/2	p150/5-6	p1254/4	p17/5-p18	p1017/3	p710/2	p3215/1	p11/1	p557/14, p558/1	p46/2
Adequate	Adequate	Adequate	Adequate	Adequate	Adequate	Adequate	Adequate	Adequate	Adequate	Adequate	Adequate
p9/3	p745/5-746	p11/2	p150/5-6	p1254/4	p17/5-p18	p1017/3	p710/2	p3215/1	p11/1, p12/2	p557/14, p558/1, p559/2-3	p46/2
plausible and appropriate answer to research question	plausible and appropriate answer to research question	plausible and appropriate answer to research question	plausible and appropriate answer to research question	plausible and appropriate answer to research question	plausible and appropriate answer to research question	plausible and appropriate answer to research question	plausible and appropriate answer to research question	plausible and appropriate answer to research question	plausible and appropriate answer to research question	plausible and appropriate answer to research question	plausible and appropriate answer to research question
Appropriate	Appropriate	Appropriate	Appropriate	Appropriate	Appropriate	Appropriate	Appropriate	Appropriate	Appropriate	Appropriate	Appropriate
p3/3	p738/6	p5/2	p146/4	p1246/4, 7	p7/6	p1012/7	p704/3	p3209/5	p3/1	p556/2	p44/3
basics available (ethics approval), could be more detailed	ethical approval and description of information that participants received	ethical approval and description of information that participants received	basics available (ethics approval), could be more detailed	ethical approval and description of information that participants received	basics available (ethics approval), could be more detailed	ethical/municipal approval and description of information that participants received	ethical approval and description of information that participants received	ethical approval and description of information that participants received	basics available (ethics approval), could be more detailed	basics available (ethics approval requested), could be more detailed	basics available (ethics approval), could be more detailed
++	++	++	++	++	++	++	++	++	++	++	++
